# Panventriculomegaly with a wide foramen of Magendie and large cisterna magna: clinical, radiological, and genetic analysis

**DOI:** 10.1186/2045-8118-12-S1-O28

**Published:** 2015-09-18

**Authors:** Hiroshi Kageyama, Ikuko Ogino, Kazuaki Shimoji, Madoka Nakajima, Ryoko Fukai, Noriko Miyake, Kenichi Nishiyama, Naomichi Matsumoto, Hajime Arai, Masakazu Miyajima

**Affiliations:** 1 Juntendo University, Japan; 2 Kuki General Hospital, Japan; 3 Yokohama City University, Japan; 4 University of Niigata, Japan

## Introduction

The purpose of this study is to provide the first clinical, radiological, and genetic analysis of panventriculomegaly (PaVM) defined by a wide foramen of Magendie and large cisterna magna.

## Methods

Clinical and radiological data from 28 PaVM patients (including 10 patients in 5 families) were retrospectively analyzed. Five children were included. Tetra-ventricular dilatation, aqueduct opening with flow void on T2 weighted images, and a wide foramen of Magendie and large cisterna magna were essential magnetic resonance imaging findings for PaVM diagnosis. Three-dimensional fast asymmetric spin echo sequences were used for visualization of membranes in basal cisterns. Time-spatial labeling inversion pulse examination was performed to analyze cerebrospinal fluid (CSF) movement. Gene mutations were analyzed using high-resolution microarray and validated by quantitative PCR with breakpoint sequencing.

## Results

In adult patients, the age of onset was 56.0 ± 16.7 years. It was lower than that of idiopathic normal pressure hydrocephalus (iNPH). Adult patients showed iNPH like symptoms, as gait disturbance, urinary dysfunction, and cognitive dysfunction. Five infantile patients exhibited macrocranium. Patients were divided into two subcategories, with or without downward bulging third ventricular floors and membranous structures in the prepontine cistern. CSF movement of patients with bulging floors, who had thick membranes in the prepontine cistern, was restricted in prepontine cisterns. Patients with bulging floors were successfully treated with endoscopic third ventriculostomy. Genetic analysis revealed a deletion in DNAH14 that encodes a dynein heavy chain protein associated with motile cilia function, and which co-segregated with patients in a family without a downward bulging third ventricular floor.

## Conclusion

Panventriculomegaly with a wide foramen of Magendie and large cisterna magna may belong to a subtype of congenital hydrocephalus with familial accumulation, younger onset, and symptoms of iNPH. In addition, a PaVM family has a gene mutation associated with dysfunction of motile cilia.
